# Strengthening and Institutionalizing the Leadership and Management Role of Frontline Nurses to Advance Universal Health Coverage in Zambia

**DOI:** 10.9745/GHSP-D-18-00067

**Published:** 2018-12-27

**Authors:** Allison Annette Foster, Marjorie Kabinga Makukula, Carolyn Moore, Nellisiwe Luyando Chizuni, Fastone Goma, Alan Myles, David Nelson

**Affiliations:** a IntraHealth International, Washington, DC, USA.; b University of Zambia, Lusaka, Zambia.; c mPowering Frontline Health Workers, Jhpiego, Washington, DC, USA. Now with FHI 360, Washington, DC, USA.; d University of Zambia, Lusaka, Zambia. Now with FHI 360, Lusaka, Zambia.; e Independent consultant, The Lee, Buckinghamshire, UK.; f IntraHealth International, Chapel Hill, NC, USA.

## Abstract

In Zambia, nurses and nurse-midwives lead more than half of rural facilities and guide primary health care delivery. Based on a formative assessment, the Ministry of Health (MOH) determined that improved leadership capacity and management skills of facility heads would help maximize the potential of Zambia's community-level investments. In support of these efforts, the Primary Health Care to Communities (PHC2C) initiative designed and tested a 12-month blended learning program for a certificate in leadership and management practice (CLMP) to build leadership and management competencies of rural facility heads, including increasing their ability to lead frontline teams and strengthening their skills and confidence in technology use. The CLMP was created with leadership from the MOH, technical guidance from the University of Zambia, and expertise from PHC2C partners IntraHealth International, Johnson & Johnson, and mPowering Frontline Health Workers. In total, 20 nurse facility heads and 5 district nurse supervisors in 20 rural facilities across 5 districts were selected to test the course content and delivery approach. A mixed-methods approach, including evaluation of facility heads' presentations on community health improvement projects, focus group discussions with community members, and key informant interviews with nurses, clinical officers, and other stakeholders, was used to assess the results. Findings suggested that the facility heads had successfully strengthened their leadership and management competencies, increased their ability to lead frontline teams, and strengthened their skills and confidence in use of technology, including using a WhatsApp community of practice for support and consultation with other colleagues, with demonstrated improvements in the quality and accessibility of services. Based on assessment results and lessons from the test intervention, the Zambian government has committed to institutionalize CLMP as a national continuing professional development program, required for nurses posted to lead rural facilities. The planning, design, and implementation of this program offer an example to other countries and global actors of how nurses empowered with competence and confidence can play a significant role in coordinating the maze of community actors and navigating the complexities of community health systems to advance primary health care and universal health coverage.

## Abstract (precis)

Through a 12-month blended learning program, nurses and nurse-midwives leading low-resource health facilities at the community level improved their capacity to engage community members, increased their ability to lead frontline teams, strengthened their skills and confidence in technology use, and optimized investments in the community health system to achieve high-quality services.

## BACKGROUND

In 2018, we celebrated the 40th anniversary of the Alma Ata Declaration and confirmed that primary health care at the community level remains the cornerstone of improved population health. To achieve Goal 3 of the Sustainable Development Goals, countries must invest resources in primary health care to advance universal health coverage.

In Zambia, nurses and nurse-midwives still lead over half of rural facilities and guide primary health care delivery in almost all facilities. Nurses ultimately need to manage their own clinical workloads and lead task shifting, task sharing, and delegation of responsibilities among facility staff, community health workers, and community volunteers. Facility staff typically include clinically trained nurses of various levels and community health assistants (CHAs)^1^—a cadre of community health workers added to the frontline team between 2010 and 2012 who have been trained by the government and put on payroll. They provide households with health sensitization, health promotion and education, counseling, and some primary care, and are supervised by the clinical staff. Community health workers also include community-based volunteers—village members who have had various levels of training, most often from external projects, have varying levels of capacity, and who provide additional support to households, including HIV peer-to-peer counseling, counseling to pregnant mothers, and escorting pregnant mothers to facilities for delivery. Other community volunteers contribute their support through neighborhood health committees.

Head nurses need the ability to assign clinical tasks to the providers best suited to execute them and assess how best to delegate tasks among frontline team members to optimize existing skills. Facility heads must also integrate CHAs into frontline health teams and maximize the impact of CHAs and other community health workers, through guidance and oversight to the full frontline team and by building bridges with the community. This relationship with the community includes engaging with them to solve health challenges, partnering with them to build awareness of disease prevention and encourage healthy lifestyles, and community members working with the facility to improve service quality, reduce morbidity and mortality, and improve population health. Some facility heads also recruit village leaders to endorse or require healthy behaviors among households and mobilize direct assistance of community members for tasks such as building and furnishing maternity homes.

The capacity of nurses to lead community health effectively will determine the quality and safety of the services delivered and returns realized. Yet nurses heading rural facilities have not received updated scopes of practice that reflect leadership activities or additional training to prepare them for the broader responsibilities that come with heading a rural facility. Further, nurses who are deployed to lead these rural facilities have not been awarded updated titles recognizing the significance of their role or commensurate salary increases.

Zambia is striving to optimize investment dollars to meet community health needs and to ensure that its community systems can sustain health initiatives within national budgets and maintain achievements toward reaching the Sustainable Development Goals. The Ministry of Health (MOH) has leveraged development funding from the United States Agency for International Development (USAID), the UK Department for International Development (DFID), the United Nations Children's Fund (UNICEF), and other donors to integrate CHAs into the MOH staff as part of its effort to improve community health and advance universal health coverage. However, the MOH and its partners have faced difficulties in assimilating CHAs with the rest of the community team. They determined that better supervisory support and management from facility heads and district supervisors was needed to maximize the potential of the CHA investment.

Nurses have reported inadequate practical training in supervisory and management skills in their preservice education. Limited access to technologies and heavy workloads at under-staffed facilities have made it hard for nurses to access continuing professional development (CPD) opportunities to strengthen management competencies. Even intermittent workshops posed a challenge because of the time nurses are away from situations where they are urgently needed. It has been shown that workshop training without follow-up fails to demonstrate sustainable results over time.^2^

Nurses have reported inadequate practical training in supervisory and management skills in their preservice education.

## PROGRAM DESCRIPTION

In support of Zambia's efforts to strengthen the community health system, the Primary Health Care to Communities (PHC2C) initiative designed and tested a blended learning program in 2016–2017 to build leadership and management competencies of rural facility heads. Guided by evidence from a 2015 formative assessment,^3^ the MOH requested that the University of Zambia (UNZA), a PHC2C partner, and the professional councils define a program to develop greater leadership and management capacity among nurses and nurse-midwives leading low-resource facilities. (See the Figure for competencies defined through the formative assessment.)

**FIGURE:**
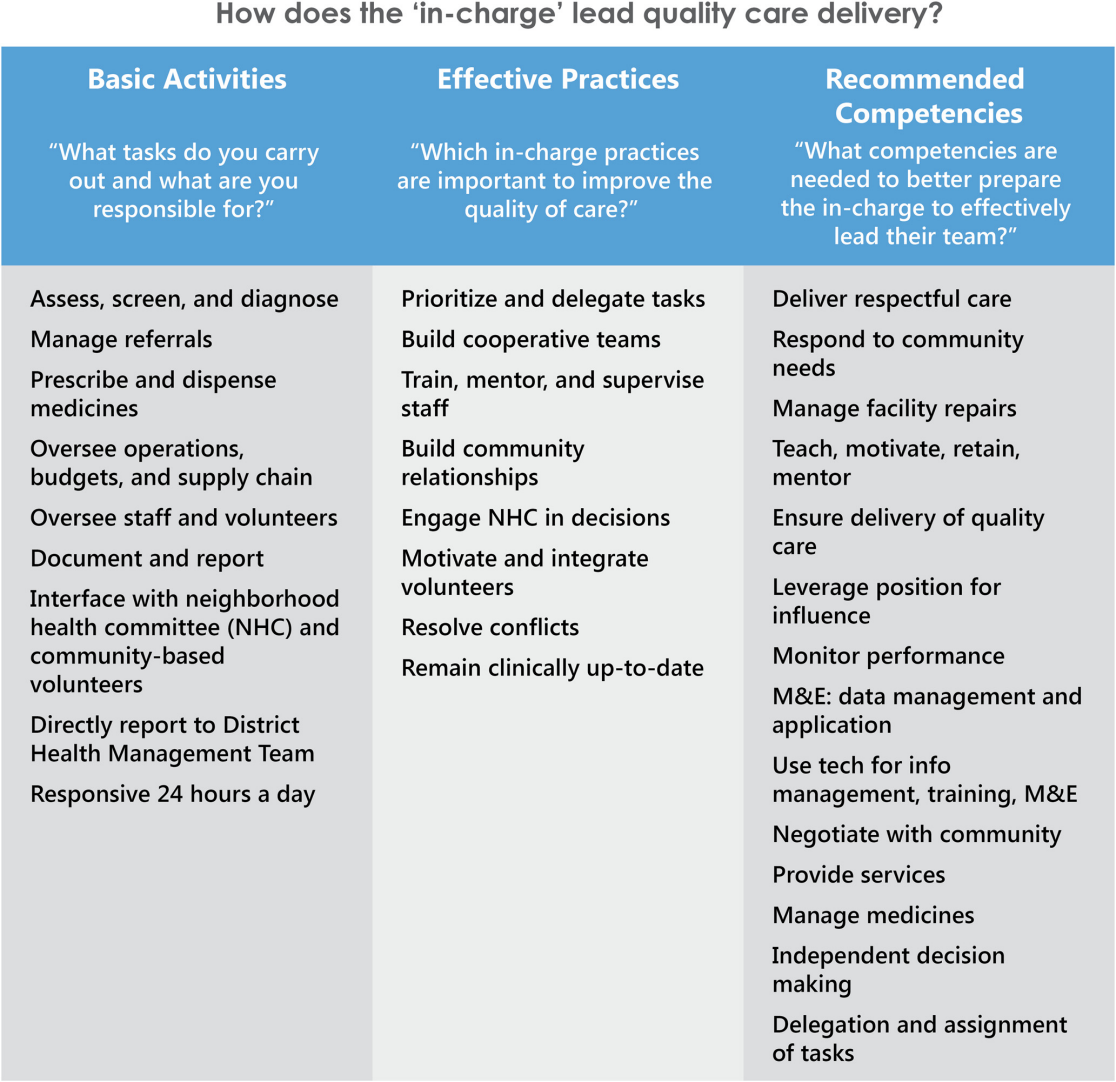
Tasks, Important Practices, and Recommended Competencies of Facility Heads Abbreviations: M&E, monitoring and evaluation; NHC, neighborhood health committee.

A blended learning program to build leadership and management competencies of rural facility heads was designed and tested in 2016–2017.

With the MOH leading the process, a competency-based, blended learning program for a certificate in leadership and management practice (CLMP) was created through technical guidance from UNZA and input from PHC2C partners IntraHealth International, Johnson & Johnson, and mPowering Frontline Health Workers. The ultimate aim of this program is to advance universal health coverage by improving the quality, accessibility, safety, equity, and utilization of care. The specific objectives of the program are to:

- Improve the leadership and management competencies of facility heads.
- Increase the ability of facility heads to lead frontline teams toward improving quality of care.
- Strengthen the skills and confidence of nurses in using technology.
- Equip nurses to better lead frontline teams in advancing universal health coverage, measured through improved service quality and access.

### Course Design

A new feature of this training, in the context of nurses working at the community level in Zambia, is its blended learning design. Short-term workshops alone lack the ongoing practice and guidance required to apply learning in context and sustain gains through changed practice. Self-guided or distance in-service programs, even those with intermittent in-person meetings or workshops, are often short-term and lack the oversight of participating supervisors. Finally, most in-service programs train one group of providers without engaging the broader team of staff and volunteers with whom they may need to work to apply new concepts and practices.

The CLMP program is designed to bring the nurses and nurse-midwives leading primary health care teams together with their district manager supervisors in a joint learning endeavor. As described in the CLMP's Facilitator's Guide (http://health-orb.org/resource/view/certificate-in-leadership-and-management-practice#files-notice), each of the in-person trainings features a pre-orientation with the district nurse managers and includes those managers in the training itself. Monthly calls, facilitated discussions, and monthly reviews of workbook exercises keep nurse managers involved in the coursework, using and reinforcing their supportive supervision roles. The aims of this inter-professional/cross-cadre training design are to:

- Strengthen supervisory and leadership skills among the supervisors while building leadership skills of the facility heads.
- Lay the groundwork for sustainability such that both management and frontline team leadership understand the same objectives and develop the same skills.
- Strengthen the relationships between the supervisors and the nurses, which are commonly weak in rural settings.
- Broaden the understanding and recognition of the potential leadership value that nurses can provide on the frontline if equipped with the training in those capacities.

Further, this joint participation in the program helps to ensure that training aligns with national policies and district guidelines and requirements, strengthening the oversight and accountability mechanisms of the national community health system.

The bulk of the learning in the 12-month program is spent in the workplace and engages community members, volunteers, and facility staff, including any additional clinical staff at the facility, CHAs, and environmental health technicians in a variety of learning exercises. These exercises are integrated with everyday work practices to ease workloads, improve efficiencies, and bring changes that facility heads, their teams, and their clients can see. The length of the program and its integration into service delivery activities allows time for new practices to become habit and perspectives and attitudes to be internalized. Course instructors for the test intervention came from UNZA and other PHC2C partners. This team led delivery of the course with ongoing input from technical advisors from the MOH, the UNZA School of Medicine, the Zambian Union of Nurses Organization (ZUNO), the General Nursing Council, and the Health Professionals Council of Zambia.

The bulk of learning in the 12-month blended learning program is spent in the workplace.

The first 9 months of the course revolve around a community health improvement project, designed so that nurses and their teams can apply knowledge and skills gained through the learning experience to achieve a goal together that improves delivery of a particular service in their community. The project is identified through consultation by the head nurse with facility staff, volunteers, and community members about which service improvement would mean the most to them. Several nurses reported that this exercise offered a fresh introduction to village leaders (e.g., village chief, council headman, council members), who were important later in supporting the work of the frontline team. Examples of projects included increasing enrollment in antenatal care (ANC), improving the quality of services for facility deliveries, and increasing uptake of testing for HIV and other sexually transmitted infections.

The first 9 months of the course revolve around a community health improvement project.

Facility staff of all cadres, including CHAs, as well as volunteers and community members join the facility heads in the development, implementation, and monitoring of the community health improvement project, with ongoing guidance from the district supervisors. These district managers, in turn, learn to provide more hands-on support and to recognize the potential of the head nurses to help them advance service indicators, community health, data management, and evidence building. The last 3 months of the program are devoted to completing the community health improvement project, evaluating the results, and preparing a final presentation for the course graduation event. The community health improvement project demonstrates the results of the training and the capacity of the facility head to build and sustain a cohesive, effective team to deliver high-quality, accessible care. The project also serves as an accountability mechanism, bringing the community and the MOH together as collectively responsible for the performance of their own health system.

The training structure incorporates peer-to-peer learning components and ongoing communication activities. As described in the CLMP Facilitator's Guide, the learning process requires that nurses use their mobile phones (all of which were owned or acquired by the course participants themselves) to participate in learning group discussions facilitated by their nurse managers during which they use WhatsApp to call their assigned “call partner” (another facility head) each week to complete that week's training assignment. This activity strengthened peer-to-peer relationships between the nurses, most of whom did not know each other prior to the course even when they were working in the same district. It also strengthened a community of practice among facility heads that had not existed before and through which nurses gain confidence not only in their own capacity as leaders in their health zone or catchment area but also in the support system they have from one another. Studies have shown that the use of social media as a part of learning improves the learning while contributing to retention of frontline health workers in low-resource posts.^4^

The training structure incorporates peer-to-peer learning components and ongoing communication activities, including a WhatsApp discussion group between nurses and managers.

The program also incorporates UNZA Mobile, a mobile application that supplements workbook reading and cross-cadre activities and reinforces learning through audio and visual tools, research links, and review exercises. UNZA Mobile is based on the OppiaMobile application and allows students to access video, audio, and text-based tools without an active network connection. When students join in-person quarterly workshops, the content for the subsequent quarter is downloaded to their UNZA Mobile app because many of the course participants live in rural areas where access to phone connections is poor. As technology and infrastructure continue to improve in Zambia, students will be able to download this content on their own. The mobile application is designed to engage students instantly and add variety to the learning process, accommodating multiple learning modalities while building self-guided research habits. Further, interaction with the mobile application is intended to raise nurses' level of self-assurance in technology use beyond social texting and voice communication.

## ASSESSMENT METHODOLOGY

Twenty facilities (rural health centers and health posts) across 5 districts in 2 provinces were selected to test the course content and delivery approach for viability of operationalization, appropriateness for uptake, and effectiveness in building leadership and management competencies in nurses, as well as to gauge how the program reinforced community and service delivery processes, health system operational mechanisms, and national and district policies. Twenty nurse facility heads (18 of whom completed the course) and 5 district nurse supervisors participated in the test cohort.

A mixed-methods approach was used to assess the results of the test intervention:

- The community health improvement projects provided the primary means of evaluating facility heads' ability to better lead frontline teams toward improving quality of care. During project presentations, results of the training were demonstrated by the head nurse's ability to describe the improvement process, identify the competencies they had used and how they had applied them, clarify contributing roles of various frontline team members, and show measurable improvement toward meeting the need or closing the gap prioritized by their project. (See Supplement 1 for the final community health improvement project presentations made by program participants and Supplement 2 for the goals and results by facility.)
- Nine months into the program, program advisors gathered with UNZA training facilitators, General Nursing Council representatives, and district managers to assess the progress of the training. Students were asked to present progress on their community health improvement projects to gauge (1) whether they had used peer-to-peer support, engaged community members to solve problems, and applied the problem-solving tools imparted during the training; and (2) how their own management skills had been employed to guide coordination of roles to accomplish tasks and move forward. The students' ability to describe the process and explain progress made was used as an indicator of autonomy and accountability, 2 leadership characteristics addressed in the course curricula. Each nurse also discussed additional areas of service improvement being pursued. Clarifying questions were posed regarding competencies gained, used, and demonstrated. (See Supplement 3 for case examples.)
- We conducted a qualitative retrospective assessment during the last quarter of the program at 18 facilities through focus group discussions and key informant interviews. A total of 54 focus group discussions were conducted (3 per facility), with a range of 13 to 20 participants in each group (neighborhood health committee members and community volunteers). Key informant interviews (n=23) were conducted with nurses, clinical officers, CHAs, and environmental health technicians working at each of the 18 facilities. The assessment focused on whether staff, volunteers, and community members had seen changes in the management practices of the facility head, increased teamwork among staff and volunteers, and improved services to clients. (See Supplement 4 for assessment questionnaires.)

## RESULTS

### Facility Heads Improved Leadership and Management Competencies

During the course's third-quarter workshop, head nurses were asked to present examples of where they were applying newly gained competencies from their training. Observations of the program instructors, validation by the district managers of the nurses' achievements to date, and the nurses' responses to unstructured interview questions suggested that the nurses had successfully gained and strengthened leadership and management competencies and were able to apply them toward achieving health outcome goals and improvement objectives. (See Supplement 3 for case examples.)

One example is the facility head's application of quality improvement practices at the Chibombo District's Mwanjuni health post. With members of her frontline team (and without direct project support), the facility head developed referral cards that CHAs and volunteers use to track the effectiveness of their counseling of women for early ANC enrollment. The CHAs and volunteers give the cards to pregnant women after visiting their homes and tell the women to bring the cards to the facility when they come for their ANC visit. The cards enable the facility head to track how many women the CHAs are visiting, cross-checking with registry information, and how many women referred are coming to the clinic. The nurse uses the cards to document which houses are being visited and which CHAs and volunteers are most effective in applying what they have learned about advocating for early ANC enrollment. It also demonstrates a low-cost solution that was feasible within the health facility's limited budget.

### Facility Heads Increased Ability to Lead Frontline Teams

The community health improvement projects showed that nurses were able to build collaboration among the different cadres in the frontline team to identify gaps, prioritize needs, plan and implement strategies to address needs, and improve the quality of care delivered to the community. Through the projects, the head nurses demonstrated their leadership as they were able to articulate their improvement goals, their SMART (specific, measurable, attainable, realistic, and timely) objectives for attaining those goals, and the indicators they defined to monitor and demonstrate improvement. As part of the presentation, the facility heads were able to explain the specific roles and responsibilities of their frontline team members in contributing to the improvement plan, and how tasks had been shifted and responsibilities shared. Facility heads articulated the leadership and management competencies they had applied through the process and how those competencies contributed to the project achievement.

Nurses were able to build collaboration among the different frontline team cadres to identify gaps, prioritize needs, plan and implement strategies, and improve the quality of care.

### Nurses Strengthened Skills and Confidence in Technology Use

Less than half of the participants owned a smartphone when they began the program. By the third in-person meeting, 90% of the participants had traded their feature phones to invest in smartphones. All students learned to download applications, access and organize files, and incorporate the WhatsApp social communications tool into work and learning.

The training participants also gained experience and confidence using computer programs. The community health improvement project required that all students develop a PowerPoint presentation to demonstrate their final results. Some nurses developed these presentations on facility or personal computers; others without access to computers wrote up their presentations and used study time allotted during the workshops to transfer their work into PowerPoint on colleagues' computers. Nurses having trouble with their presentations received help from their supervisor and peers. At the outset of the training, only 2 participants were comfortable using PowerPoint. By the third in-person meeting, only 2 were still unable to use the computer programs and functions on their own. The rest of the students, at varying speeds, were able to maneuver through presentation slides and apply shortcuts to update tables and graphs.

All of the students were comfortable using mobile phones to call and text, but initially only a few used their mobile phones to connect with colleagues for support and consultation. However, the nurses soon began to use the WhatsApp community of practice not only to fulfill their course requirement to discuss weekly questions posed by their district managers and socialize with each other but also as a network of support for their work. On at least 2 occasions, a nurse faced a difficult health care challenge while alone in their facility. Without any other support available, the nurse in distress reached out to the community of practice and found technical guidance and moral support to steer through the necessary steps in responding to the critical health need. One of these times, a woman arrived at a facility in the middle of the night with obstructed labor, and the enrolled nurse was the only health care provider immediately available. The second event occurred when a man was brought to a facility unconscious and there was no evidence as to the cause or the needed response. In both cases the patient was successfully treated due to the network of colleagues that were ready to provide their expertise through the community of practice.

### Nurses Enhanced Capacity to Lead Teams to Advance Universal Health Coverage

The community health improvement project presentations and findings from the qualitative retrospective assessment indicated that communities where head nurses had completed the training saw specific improvements in the quality and accessibility of services—2 of the 4 characteristics of universal health coverage as defined by the World Health Organization.^5^ Examples of measurable improvements reported for individual facilities through the projects included (1) increasing testing of HIV-exposed infants at 18 months from 60% to 83%; (2) increasing ANC coverage before 14 weeks from 35% to 62%; and (3) increasing the number of fully immunized children under age 1 from 6% to 80%. (See Supplement 2 for a table of project goals and results by facility.)

Examples of measurable improvements reported for facilities included increasing testing of HIV-exposed infants at 18 months, increasing ANC coverage before 14 weeks, and increasing the number of fully immunized children under age 1.

Efficiency of service delivery is 1 of 6 domains of quality care, as described by the Institute of Medicine.^6^ Toward efficiency, nurses reported delegation and shifting of tasks as one of their main learnings in describing the competencies they applied in their community health improvement projects. Over and over again, nurses told how the training had empowered them to work better with the members of the team, inside and outside their facility, to divide the work among everyone and to assign roles and responsibilities. Further, the PHC2C team coordinated with the Clinton Health Access Initiative, which had developed a training for CHA supervisors to better guide CHAs on filling out reports and following the government's CHA policies. As the MOH had invested in both programs, PHC2C worked with the MOH and Clinton Health Access Initiative to incorporate elements of the CHA supervisor training into the CLMP to maximize and sustain the benefits of both trainings.

Nurses reported their appreciation of integrating training on reporting skills and CHA policies with training on how to be more effective supervisors through coaching, mentoring, and teaching. As described by the nurses, their improved ability to shift tasks among the team members and provide quality and safety oversight enabled them, as clinically trained providers, to use their time more wisely, see more clients, and achieve population health targets. Community members and facility staff echoed this improvement as a result of the training and noted their own increased motivation and engagement with a clear role that had meaning in contributing to the health of their community.

*We teach and mentor now with the community-based volunteers and they can't just keep quiet anymore. If they see something wrong in the community, they will come to us and say, “We don't know how you're going to handle this but there is such a case in our community.” —Community health assistant, Kabweza Rural Health Post*

Timely services is another Institute of Medicine domain of high-quality care. Clients from Mwanjuni, Momboshi, and Chisamba facilities specifically noted shorter wait times and a more effectively run facility due to delegation of duties by the head nurse to the frontline team.

*The community is happy with the services because of the way we are working. It is better this year than last year because [now] they don't wait long to come and register for antenatal [care] and a lot have given birth at the clinic. —Neighborhood health committee member, Mugurameno District*

Accessibility to respectful care and high-quality services is increased by shorter wait times, improved services, and greater confidence of the community in the facility. Confidence of clients demonstrates patient-centered care, which translates not only into the care that the community member receives at the point of service but also in the inclusion and engagement of community members in improving the quality of care. Community-based volunteers and neighborhood health committee members described a clear increase in their own collaboration and the nurses' increased outreach to the community, evidenced by the nurses coming into the community physically and inviting community members to share their thoughts and participate in plans to improve services. Staff described that the head nurse shared coaching and mentoring techniques to use with clients and community members and reported that using these techniques had built more trust and confidence in the community to come to them with problems.

During focus group discussions, community members described changes they had seen in the way that nurses and their staff treated clients. For example, a community volunteer from the Kafue Mission Health Center told how the nurse explained to the volunteers that if they wanted to improve care, they needed to listen to the people they were caring for. The nurse wanted to better understand why women were not coming to enroll in antenatal care when they knew they were pregnant. She went into the community and sat with women to listen to their concerns. What she found was that some women were afraid to register with the clinic because the father was not their husband, and they thought they would be required to name the father. The head nurse then instructed all volunteers to be sure to explain to the community members that when they come to enroll in ANC, they do not have to be married or name the father. They can feel safe.

During the formative assessment, some clients had complained that head nurses and staff shouted at patients, treated them with disrespect, and never listened to their problems. In the post-training retrospective assessment, community members noted specifically that they had seen improvements in the way that the head nurse and the other facility providers communicated with clients.

*In the past nurses used to shout at the patients, but this time there is nothing; and the community appreciates that. The staff here has been told not to shout at the patients because they came to serve the community. And this improvement, we saw it this year. —Neighborhood health committee member, Chisamba Rural Health Center*

## DISCUSSION

The CLMP program is designed to be sustainable in several aspects. The job requirements of the district supervisor include the supervision and coaching of facility heads and participation in the program, for which they also earn CPD points. The training allows nurses and nurse-midwives to fulfill their relicensure requirements with the council, and to gain the additional title of head nurse in charge. Environmental health technicians and clinical officers are also motivated to pursue the course as their councils recognize it for their CPD units. The MOH recognizes the need and value of improved leadership and management capacity to strengthen primary care delivery and build stronger community systems.

### Key Factors for Optimizing Success

The planning, design, and implementation of this program may offer an example to other countries and global actors of how investments in community systems can be optimized and global goals can be realized in local contexts, and of the commitment and accountability that is required to foster sustainable return on investments. Key factors for success follow.

#### Country Ownership

The intervention met a country need and responded to priorities defined by health authorities and recognized by relevant actors.

#### Country–Community Alignment

Community engagement is an important part of successful health system designs that respond to community needs and advance health-seeking behaviors. A variety of interventions have been implemented to increase and institutionalize community engagement, including social accountability mechanisms, neighborhood committees, quality assurance, and community development groups. We designed this intervention to reflect community voices and engaged community input throughout the process to ensure responsiveness to community needs and community requests for improvement. The program helps to build skills and expectations in the community on how to interact with health facility staff as part of routine work practices and see themselves as part of the frontline team accountable for service quality and health outcomes. It also builds skills and practices among the nurses for collaborating with community leaders and integrating community contributions within the roles and responsibilities of their teams. Further, key stakeholder participation—namely district and provincial management, professional councils, and unions and associations—bridged the gap between community voices and central government awareness.

#### Scale

The approach to this program, from its initial development to its implementation, was designed to enable institutionalization at a national level. Partners, both those leading in the country and those providing peripheral experience or resources, followed development principles that were inclusive, evidence-based, contextualized, viable, and flexible:

- **Inclusive:** Key stakeholders, from the community to the government, were engaged from the initial verification of evidence to the interpretation and application of that evidence to recommendations and decision making.
- **Evidence-based:** Evidence-based strategies ensured that the interventions were designed to fit the need and that indicators monitored reflected the expectations of the communities served and quality standards of the health system.
- **Contextualized:** The intervention was shaped to respond to the national infrastructure, health systems mechanisms, and cultural mores of the environment.
- **Viable:** Tools, mechanisms, and training delivery design were all integrated within systems structures, practices, hierarchies, and resources. Succession of program ownership was factored in from the beginning so that removing external support did not end the program.
- **Flexible:** Internal and external partners collaborating on program development remained flexible in applying previous experience and expectations so that individual or even collective agendas would not obstruct needs to adapt the program throughout its initial testing.

At the end of the test intervention in 2017, the MOH approved scaling up the CLMP program, with financial support, and linked CLMP completion and certification to an elevated title for nurses and nurse-midwives. The MOH has also reorganized the establishment of positions and salaries, although the rural facility “in-charge” position is not yet funded by the Public Service Management Division. As of this writing, ZUNO is negotiating for a salary increase upon CLMP completion and assumption of increased leadership responsibilities.

At the end of the test intervention, the MOH approved scaling up the blended learning program.

Based on the learning from this experience, the MOH has encouraged all local institutions to provide in-service leadership training for nurses and midwives working in rural facilities, and it has approved the CLMP as a national CPD program, required for all nurses posted to lead primary health care services at rural health facilities (centers and posts). Institutionalization is being led through UNZA as part of its CPD offerings for relicensure, to be sustained through student payments and annual district budget funds earmarked for CPD. UNZA is also seeking to reduce implementation costs through support from stakeholders such as ZUNO. In January 2019, UNZA will roll out the CLMP to 3 additional provinces—Eastern, Luapula, and North Western—targeting 25 to 30 rural facilities in each where service quality indicators are low. ZUNO has committed financial support to supplement existing provincial support for students who need assistance to take the training.

In addition, the UNZA School of Nursing Sciences has incorporated elements of the CLMP into its updated preservice curriculum for the Bachelor of Science degree program in public health nursing to strengthen the leadership and management component. An updated curriculum, including a community engagement component and community health improvement project model, is planned for implementation by the 2019 academic year. The School of Nursing Sciences hopes to link the improved preservice curricula more closely to CPD with the support of the MOH.

#### Maximizing and Optimizing Resources

Throughout the planning, development, and implementation of the program, country leadership guided budget decisions so that all program elements would remain financially viable within the national resource structure. The existing management structure provided mentoring and supervision of the nurses during their work, without additional support from the project. Phones and computers were not provided. To make it easier in the future to provide closer supervision of students, preceptors from referral hospitals have been added to the design. The preceptors, students who are completing their degrees during their in-service clinical practice, will receive credit toward the completion of their course by facilitating the online community of practice discussions, participating in the bi-monthly nurse-supervisor calls, and checking workbook exercises. This additional layer of support will not cost the MOH or students, but it will help to give preceptors broader supervisory experience while gaining course credit, and will provide additional on-the-ground mentoring in between the nurses' monthly check-ins with the district managers.

The program was also designed to complement other implementation projects going on in the catchment areas. For example, we aligned training on documentation and reporting with new guidelines on how reporting should be done with CHAs and the greater participation of neighborhood health committees and incorporated the MOH's CHA supervisor training in our supervision, teaching, coaching, and mentoring module. Another key measurement of success was the program's contribution to helping community actors sustain and advance the improvements made in their own communities. For example, nurses applied their skills to advance the MOH program to expand maternity homes as part of Zambia's plan to increase facility deliveries. In one case, the facility head used her negotiation skills to recruit the village leaders' support in galvanizing contributions from community members to complete the facility's maternity home, which had been delayed due to limited resources. In presenting this work in her community health improvement project, the facility head described how she had employed some of the approaches learned through the CLMP for mobilizing resources and combined that skill with some of the tools learned for community engagement to assume responsibility for her own facility and demonstrate some autonomy and leadership in achieving the goal even without the help of external funds or district intervention.

#### Holistic Approach to Capacity Building

Although this program aims to develop competencies in one cadre—nurses or nurse-midwives leading low-resource facilities—it is also designed to build the skills and knowledge of actors across the frontline team, including community members. Program developers understood that vertical interventions focusing on a specific disease or system can create imbalances in the system. Therefore, the intervention was designed to be holistic and to strengthen the system's capacity horizontally by strengthening roles for nurses in leadership and management.

### Limitations

The small sample size of the test intervention limits the reliable applicability of the results across larger populations, and the scope and 12-month length of the intervention precluded assessment of longer-term quantitative improvements in services. While some community health improvement projects documented measurable improvements in HIV/AIDS, maternal and child health, or sexual and reproductive health services, these cannot be considered significant until continued or stabilized over an additional 12 months.

## IMPLICATIONS FOR REPLICATION

Designing a fit-for-purpose workforce, meaning a well-integrated team of health workers that is prepared to respond to the needs of the population it serves, requires that countries remain flexible and responsive in education and training. Building capacity stretches far beyond preservice education and in-service training—it is a continuous journey in which various actors in the health system must learn to work together and supervision and management must support professional growth, development, and advancement. Building these skills and attitudes must start in preservice education, by establishing an understanding among clinical professionals of their roles as leaders and managers.

It is in these roles that providers can move services further toward patient-centered care, using the soft skills that are so essential in building responsive, accountable teams and are borne through engagement and interdependence among the clinical providers, the community health workers, and the community population. As Odugleh-Koluv and Parrish-Sprowl^7^ advocate:

*Health systems and communities are in continuous and interdependent action. If community engagement becomes a focus for UHC efforts, it could finally push the health sector from an almost exclusively transactional model into one that recognizes that health and well-being are co-produced, and that empowers both health-care providers and communities. This means that governments, donors and researchers have to address institutional culture and invest in so-called soft skills …*

In its approach to community health, Zambia may provide an example for other countries of the region and beyond. When developing strategies to leverage community health workers and strengthen the community health system, it has considered the entire system and how a fit-for-purpose workforce best brings together communities, volunteers, and health facility staff as a cohesive frontline team. Using existing resources and working within national systems and mechanisms, the Zambian government is committed to institutionalizing a program that will continue to strengthen leadership among community providers, build sustainability and resilience within the community system, and improve services toward achieving Sustainable Development Goal 3. Maximizing potential of community-level leadership will go a long way toward realizing people-centered, accountable community health systems that provide access to high-quality care for all.

## Supplementary Material

18-00067-Nelson-Supplement3.pdf

18-00067-Nelson-Supplement1.pdf

18-00067-Nelson-Supplement4.pdf

18-00067-Nelson-Supplement2.pdf

## Notes

**Peer Reviewed**

**Funding:** Johnson & Johnson.
