# Supplementary material for: Strengthening and Institutionalizing the Leadership and Management Role of Frontline Nurses to Advance Universal Health Coverage in Zambia
Source: Glob Health Sci Pract. 2018 Dec 27;6(4):736–46. doi: 10.9745/GHSP-D-18-00067 (PMC6370361; doi:10.9745/GHSP-D-18-00067)
Supplement: 18-00067-Nelson-Supplement3.pdf [file 18-00067-Nelson-Supplement3.pdf]

CASE EXAMPLES OF COMPETENCY DEVELOPMENT

February 2017

Training Module	Competency	Case Example of Competency Development	
		Initial Status	Improved Status
**I. Leadership and Management**			
Management	Strategic Planning		
		• **No experience defining own goals.** Only three of the facilities' heads (FHs) were able to clearly define an improvement goal to address an identified need or gap. They had followed project plans and district plans, but had not been accountable for their own plans.<br><br>• **Lack of understanding of measurable objectives**. None of the facilities heads were able to construct SMART objectives at the beginning of the course, even though they claimed to understand the concept.<br><br>• **No experience in designing work plans toward reaching goals.** None of the facilities heads had had the experience of breaking goals into weekly product-oriented activities in order to schedule and monitor progress toward objectives	**Defined accountable goals.** Using their Community Health Improvement Project (CHIP) guide, facilities heads were required to assess their community and determine their priority improvement goal with their community, volunteers, and staff. Each FH defined his or her goal and made a commitment to the frontline team members and the community make progress together toward those goals over the next 12 months.<br><br>**Constructed SMART objectives to reach goal.** Although in theory the FHs claimed to understand SMART goals, only one of the FHs was able to define a measurable, timed goal at the beginning of the course. By the 9-month workshop, each FH successfully presented SMART objectives to achieve their CHIP goals.<br><br>**Combining process-oriented activities and product-oriented actions to reach goals.** Initial activities designed to achieve objectives were primarily process-oriented (for example, 'meet with staff'). The management instruction emphasized the importance of activities that are aimed to result in tangible outputs in strategic planning. FHs returned to third workshop with both process- and product-oriented activities (i.e.: 'meet with staff to assign roles for each frontline team member').<br><br>**Scheduled objective-related activities into weekly agenda.** Creating an accountability structure, FHs integrated the activities toward meeting their objectives into their calendars along with their weekly meetings, clinical outreach, training workshops, and PHC2C workbook assignments. The district supervisors reported that the FHs had never had to do this on their own initiative prior to the UNZA PHC2C training.
	Management of physical resources		

		• **Need better management of commodities flow.** The FHs had management systems to accurately plan and order commodities used in the facility. However, monitoring the needs in the broader community needed improvement.<br><br>• **Facilities must upgrade their environments (cleanliness, safety, repairs)**<br><br>• **Need to mobilize resources from external sources.** Facilities have limited resources, and funding falls below budget requirements. Part of the UNZA PHC2C training requires FH's to find new sources for facility supplies, clinical commodities, and services to maintain and upkeep the facility structure. Two of the FHs reported that prior to the training, they had tracked needs and completed requisitions with matching budgets; but they had not stretched beyond the standard processes to use creative problem-solving, decision-making, or negotiation to expand services, procure additional resources, or recruit assistance from vendors.	**Monitoring commodities needs**. One FH had increased HIV prevention outreach as part of her CHIP goal. Even by the second quarter of the course, the facility needed additional condoms due to effective outreach efforts. To anticipate changing commodities needs and adjust orders and budgets accurately, the FH designed a simple tracking system with timed reviews to monitor use and guide commodities requisition processes.<br><br>Increased emphasis on facility maintenance as an element of quality care: Several facility heads had quality improvement goals for their facilities, and these goals included improving cleanliness, privacy, and other elements related to the facility.<br><br>**Creative mobilization of resources by recruiting local NGOs and private sector to fill resource gaps.** Two facilities leveraged projects or organizations working in their districts, unrelated to their improvement project, to gain extra support to achieve their CHIP goals. The Momboshi FH negotiated with the Tokushima International Cooperation Organization (TICO), a Japanese NGO, to help them in training SMAGS and providing supplies to the facility to enhance institutional deliveries. A second example where FHs stretched their own ingenuity to recruit resources is in the Kafue Mission RHC where the FH lobbied for external help improving their antenatal care services and succeed in recruiting hemoglobin testing kits from the Center for Infectious Disease Research in Zambia (CIDRZ) and obtained treated mosquito nets from the Child Fund. The FH was successful in both negotiations, and reported to the group that she had used the 'skilled influencing' methods practiced in her training exercises. She added that she had been surprised at her own ability to creatively leverage support from new sources.
	Management of human resources		
	Build Teams	• **Village heads had not been active in the health improvement efforts.** Although the facilities heads had had interaction with community leaders, none of the facilities	**Recruit the active participation of village headmen.** Several of the facilities heads challenged themselves, through the training exercises, to use influencing communication techniques to engage more of the community, and even the village headmen into frontline team efforts. One example is in the Momboshi catchment area, where the FH travelled to

		heads had gone out into the community to lead their own assessment, and to engage the community members and leaders in a conversation about their needs. The assessment included in the UNZA PHC2C training requires the FHs to ask, listen, suggest, and request. FHs have typically not been trained to take that extra step or go the extra mile to reach out to community members and leaders to engage them in conversation and in commitment to shared action.<br><br>• **FHs and CHAs alike had reported the lack of clarity around roles of the new staff cadre of community health assistants (CHAs) should play and around their interface with volunteers and other members of staff**. Facilities heads are accustomed to 'meet the moment,' assigning tasks and responsibilities to staff and volunteers on hand. Because rural facilities depend heavily on volunteers that have varying schedules, part time staff (as Environmental Health Technicians and Community Health Assistants work only part time at the facility site), FHs need to identify what strengths and weaknesses and be able to delegate tasks accordingly on any given day with the providers that are available. More importantly, galvanizing the frontline team to work collaboratively toward specific goals, the FH must assign clear roles and	the villages to visit directly with the village headmen and ask for their support in improving MCH services to the community.[a] The village headmen supported the FH's request by announcing in their villages that women were expected to go to facilities for deliveries and that village council leaders should help pregnant women to overcome any obstacles to facility deliveries. The village headmen of one village even set a policy that households would be fined for home deliveries. Several FHs commented that they had previously not considered this type of community engagement as part of their role.<br><br>**Expanding the role of neighbourhood health committees and broader local community; and the leadership of the FH**. Even though Neighbourhood Health Committees (NHCs) had been established by the MoH and partners, the role that the NHC members had been limited. FHs reported that members would come to facility each month, bring monthly report forms with information updates from the community (reports of illnesses, pregnancies, or concerns), and receive information to take back to the community. Through this program, all 9 of the FHs gave specific examples of how they had expanded the role of the NHC members and engaged their active contribution to improving services and achieving the CHIP goals. The head of the Rufunsa RHC learned that, despite working with her CBVs to advocate facility deliveries, there were still a large number of mothers living in distant villages delivering at home. The primary reason that the villagers gave for the low facility delivery (34%) was the lack of transportation. The district health office had planned to build a mother's home to facilitate institutional deliveries for village women living far from the facility; however, the plans had been stalled due to lack of district resources.<br><br>The facility head presented the challenge to her frontline team, including civic leaders the local Zambia Rufunsa office of the Police Department and the NHCs, and they understood that without help from the community, the maternal home would not be built. Through the advocacy and negotiation of the facility head, the civic leaders donated building blocks and the NHC members raised money from the villagers to transport the blocks to the building site. Donations from the community also included bedding materials for the women who would come to stay once the building was complete.

[a] Village headmen do not typically engage with the health system directly or engage with facilities heads. Facility heads, and district managers as well, will go to village heads to obtain permission to enter the village or to implement a project in their village, but it is not a usual practice to involve the headmen in roles to improve health services for the community. In some districts in Zambia, projects have engaged village leaders in water sanitation efforts and other support, but for these facility heads, the partnering with village heads is a new practice, which, from their reports, is strengthening their efforts.

		responsibilities. FHs had not taken a systemized approach to integrate new staff members (CHAs), volunteers and community members from NHCs with clear roles and accountability for performance. Many FHs were uncertain of the scope of work of CHAs, and some did not know whether the CHAs were intended to report to the FHs or to the district.	When the Provincial and District management teams observed the commitment of the village members to improve the health of their own community, they contacted UNICEF through the Millennium Development Goals Initiative (MDGI) programme and requested assistance for the last amount of funds to build on what the villagers had achieved and fund the construction of the maternity ward and mother's waiting home. The strengthened leadership of the head nurse across all members of her frontline team solved a seemingly insurmountable challenge and inspired others to step in and help as well.<br><br>The FH of the Shiyala RHP in the Chongwe District reported that before the training, it had not been clear to her or to her team how the CHA was supposed to contribute to the facility, what reporting structure they were to follow, or how much time the CHA should spend in the facility. After the adapted and combined PHC2C and CHAI trainings during the second workshop, the facility head met with the CHAs and the rest of the team members to explain to the CHAs roles and responsibilities and to adjust the reporting system at the facility. The facility head also reported that because they had clarified a role for the CHAs, she delegated supervision of some of the CBVs to the CHA, and then instructed the CHA to report on their CBVs progress in their assigned area of the community or zone. The facility head further explained that the clarity and guidance in both the official roles of the CHAs and in effective approaches to supervision and mentoring, the CHAs responded positively and there was higher level of engagement and positive attitude among them and the CBVs because everyone felt appreciated for his or her contribution. Several FHs reported that the combined PHC2C and CHAI training had improved their ability to work with CHAs, delegate tasks to CHAs, and mentor CHAs.
Leadership	Respond to community needs; Influence change; make independent decisions	**Need for empowerment to meet community needs.** Meeting community needs is not only a standard WHO leadership competency but also a characteristic that community members defined as critical to a head nurses' ability to deliver quality care. Communities further defined that characteristic as listening to the community and understanding what they identify as their needs. Communities suggested that they would prefer to participate in prioritizing service needs rather than being told what priorities had been defined for them.	**Head nurse initiates new Family Planning services to meet community need.** Upon a community assessment, which is the first stage of UNZA's PHC2C training, the FH from the Chikoka RHP in the Kafue District discovered a significant unmet community need for reproductive health services. At that time, the post did not offer family planning (FP) services, so Ms. Kufekisa and the members of her frontline team committed to add FP services that would be accessible to women and adolescents. The head nurse trained CBVs in FP counseling and methods, and requisitioned condoms for the CBVs to distribute among the villages. The head nurse also trained the CHAs and other facility staff and continued to coach them over time to improve their approaches in counseling women on FP choices, offering different products, and

			providing on-going emotional support. She added FP products to the facility budget and incorporated various products to the commodities requests. In addition, with input from her frontline team, the FH created check lists to guide staff and volunteers to consistently deliver quality FP services.
	Market Services	**FHs had not engaged in marketing their services**. Prior to the training the reported that they did not understand the concept and did not know how to 'market'. Many knew that their community members travelled to other facilities outside their catchment area or complained about the services that their own facilities provided. None of the FHs had thought about the importance combining the improvement of services with the alerting of households so they would return to the facility to try the improvements or expansions.	**Combining improvements with marketing.** The FH from the Chikoka RHP, who had initiated FP services in her facility, designed promotional activities with the members of the NHC, which they implemented not only to raise awareness of the benefits of family planning, but also to market the post's new services and attract women and youth to the facility. The head nurse, in reporting to the group of her progress, pointed out that she had used some of the guidelines gained in her training to craft 'marketing messages' to disseminate to through the community. Over time the FH hopes that her monitoring will show increased utilization of the facility.
**II. Quality Improvement (QI)**			
	Identify gaps; design solutions; test solutions; manage Data; Use data for monitoring and evaluation; apply evidence to decisions	***Lack of real quality improvement application.*** At the beginning of the course, FHs, and even the district supervisors, were familiar with general QI theory and principles; however, they were not able to apply those theories to practice. None of the FHs, and only two of the five district nurse managers were able to define measurable indicators for objectives. In addition, when improving services in the field, FHs reported that it was difficult to track the effectiveness and service quality of their volunteers and CHAs working away from the facility and in the villages. Evidenced from the research and from the experience with the course participants, there is a clear need for quality improvement application and practice for facilities and their communities to lead their own service improvements and move forward toward better health.	**Designing referral cards to track effectiveness of CHA and CBV counseling.** Through the exercises of the workbook, the FHs were asked to document the effects of their interventions toward reaching the objectives of their CHIP goal. The FH from the Chibombo district's Mwanjuni RHP created a referral card to help him track where CBVs and CHAs were advocating early enrollment to ANC. The facility head, along with the community, had identified early ANC enrollment as their CHIP goal, and one of the objectives was toward reaching that goal was to engage the CBVs and CHAS in direct house-to-house advocacy. To understand how that advocacy was affecting the number of women coming to the facility (and test their assumptions), the team developed referral cards that the CBVs and CHAs would give to the pregnant women after visiting their home. The pregnant women then brought these cards to the facility when they came for their ANC visit. Counting the cards and tracking their origin achieved two monitoring and evaluation objectives: first the FH could count how many of the pregnant women had come due to the visit of the newly trained CHA and CBV. Second, the FH could see which houses and were being visited and which CBVs and CHAs were most effective in applying what they'd learned. Making the card even more useful, the facility staff then signs and dates the card after the ANC visit, so that when the CHA or CBV next visits the household, they know that, in fact, the pregnant woman followed their counseling and enrolled in ANC. In addition, if the woman were to go to another facility for the

			next ANC visit, that facility would also see that the woman had enrolled in ANC, and would know when she had last received care. Even though this innovation is simple and 'low-tech' the facility head demonstrated creative problem-solving and an understanding of quality improvement practices. Further, she established a practice in her facility that would enhance and improve ANC enrollment and follow-up.<br><br>In general, the FHs demonstrated improved ability to track service provision and outcome data, and interpret that data into action plans. One interesting example came from a FH who was working to improve STI contact tracing in her community. While her ultimate goal was to reduce the incidence of STIs, she noticed an increase in STI incidence based on clients coming to the facility. She understood that this information, rather than showing a negative trend, indicated an improvement of contact tracing and community members with STIs seeking treatment.
III. Evidence Based Practice			
Quality Clinical Services	Provide quality clinical services	The evidence-based practice module of the four-module training is the most flexible. It is intended to incorporate instruction on new products, new protocols, refresh earlier training or pre-service instruction, and provide guidance for emergency response. UNZA harmonized efforts with the MoH to use leverage the training to address new MoH protocols within the EBP module.	**New protocols for HIV/AIDS treatment**. One example of a contextual application of this module was when UNZA and PHC2C partners agreed with the MoH to incorporate new guidelines on the HIV/AIDS treatment into the module. Training in the guidelines was linked to the workbook exercises, and supervisors who inspected the procedures reported that all facilities had successfully incorporated the new guidelines into their practices. By incorporating new guidelines or procedures into the course work, the MoH avoids additional workshops and also integrates the new practices and supervision into their on-going work at the facility.<br><br>**Emphasizing adolescent services**. A second example of incorporating EBP into the course is with the new guidelines for adolescent health. Again, UNZA incorporated the guidelines into the workbook materials with exercises to practice and test the implementation of the guidelines. Supervisors reported that three facilities, Chikoka, Rufunsa and Mwanjuni, had all initiated adolescent- and youth-friendly services in their facilities.
	Deliver respectful care	During interviews with patients and community members three of the 20 facilities scored low in patient satisfaction. Even though the other facilities scored high, qualitative data revealed the concerns of both patients and community members regarding disrespectful care. The lack of respect described ranged from lack of attention, tardy arrival, and lack of privacy to more aggressive yelling and name-calling.	***No evidence of improvements in reports from FH in third workshop***

**IV. Supervision, Coaching, Mentoring, & Teaching**			
	Supervision: Supervise and encourage optimum performance of providers both inside the facility and outside in the community; Supervision styles to support quality care from staff, volunteers, community members and even community leaders.	**Filling a need for stronger supervisory support from the district management team.** Supportive supervision has been shown to improve performance, motivation, and retention of health providers. One of the benefits from the training design is that the supervisors mentoring the FH students improved their own supervisory practices while they helped the FHs to improve theirs.	**Supervision improved between district level and facility level**. FHs and supervisors alike reported that, prior to the training, they had only communicated with each other when the supervisor needed information from the facility or during the performance assessment. Supervisors and facilities heads agreed that the training had opened new avenues of communication between them, which were continuing outside the boundaries of the training assignments. Both groups felt comfortable to call each other on the phone to ask a question or to share information. The supervisors said that they now saw the facilities heads as individual people rather than just positions in the field, which helped them to provide better personalized guidance. Further, the supervisors reported that they valued the FHs more highly than they had previously, realizing that they could delegate responsibilities to them and seek useful input for improvement strategies.
	Coaching: Provide on-going guidance and encouragement with staff and volunteers toward improved service delivery, and with community members to adapt healthy behaviors; Peer to peer coaching among facilities heads to respond to community needs.	**Peer-to peer coaching among FHs to best respond to clinical emergencies**. FHs working in remote areas often work alone. Even when they have staff and volunteers, they may still need the support of other trained FH's when responding to complex situations. The FHs of this course only had their district nurse supervisor to rely on for experienced input and support. However, the nurse supervisor may be unavailable or far away.	**Peer-to-peer coaching saves a woman in labor**. A pregnant woman arrived in the middle of the night at the Kabweza RHP in distressed labor. The FH was not a midwife and was unsure of how to respond to the situation. Reaching out to the community of practice, the FH tapped into help from other nurses and midwives, who guided him step by step, Facilitators of the course, living in Lusaka and Washington DC were captivated for the hour-long exchange between more experienced nurses and the FH, as the drama unfolded and the FH was coached through the tense moments. In the end, colleagues were coached the FH through the right decisions to identify an obstructed labor, respond accordingly, and safely transport the patient to the nearest referral facility.
Mentoring	***Mentoring:*** Provide assistance in expanding or improving competencies; Provide career guidance		***No evidence of this yet.***
	Teaching:	**Need for stronger role as teacher and coach**.	**FH expands STI services by teaching new skills to frontline team.** The

	Instruct frontline team members in clinical protocols and practices and educate community members	FHs agreed with the research findings that they needed to strengthen their teaching role by taking responsibility to expand and improve the skills of their staff and volunteers. The training exercises challenge the FHs to build skills of their frontline teams as a means to improve services as well as motivate providers.	head of the Shiyala RHP in the Chongwe District responded to community needs for broader STI counseling and testing. STIs were a growing concern for the community. Only a very few of the CBVs had been trained as peer counselors to provide counseling and testing for STIs. The facility head assumed accountability for responding to the need for access to STI services. She trained all staff, including CHAs, and additional CBVs to be able to give both counseling and testing for STI's. Because they were new at this responsibility, the facility head reported that she met with different individuals regularly to observe their practice, give feedback, and talk with them about challenges or concerns in their new roles. The facility head said she felt she had effectively responded to a community need by overcoming an obstacle to access to reproductive health services through several management and leadership practices, including assigning clear roles, teaching new skills, coaching, raising performance expectations, and being creative in problem solving.
**V. Use of Technologies**			
	Proficiency in technologies that facilitate work toward greater efficiency, productivity, and effectiveness	**Lack of familiarity, competence and confidence in technology use.** The 2015 formative assessment identified 'proficiency in technology use' as a significant in the necessary leadership competencies of an effective facility head. The UNZA PHC2C Leadership and Management training does not dedicate a module specifically to this topic, but rather weaves technology use throughout the course so that participants learn to incorporate mobile phone apps and computer programs into their work activities, thereby increasing their confidence and proficiency.	**Understanding Mobile Apps**. Mobile applications abound in the community health space. The UNZA PHC2C program offered the facility heads additional understanding of what mobile applications are, and how to download and access them During their workshops, participants downloaded content - on either SD cards from a computer or directly onto their phones from the internet - which they could later access online. FHs also learned how to find and manipulate mobile phone files once the content had been uploaded. Four FHs claimed to be confident and proficient with mobile phone apps and software, and one of those four assigned himself as the coach for the others, for whom these skills were new. As part of the program, an 'UNZA app' was designed by Digital Campus, IntraHealth, mPowering, and UNZA. The course requires that the FH student use the app in conjunction with course material, allowing students the experience of finding literature resources even in locations where there is no internet connection, accessing video instruction audio re-enforcements, and interacting with self-guided tests to review their learning. Managing the app helped students to reach beyond the '*WhatsApp*' social media application and the limited spectrum of use they were familiar with to gain curiosity and confidence in manipulating information with their 'UNZA app,' which prepares them for internet access that will come in the future.<br><br>**Using power point applications**. The facilities heads from the most rural areas, such as Muguramemo and Kabangalala reported that they had never used a computer. Of those who were familiar with computers, only three (3) had created a power point presentation or presented it. The

			UNZA PHC2C course requires the facilities heads report on their Community Health Improvement Projects (CHIPs) through a power point presentation. At the beginning of the course only two FHs were using computers and many had no access to a computer. Even though their facilities have all been equipped with solar powered electricity, they are still relying on paper-based systems and pencils and paper to document and record. By the third workshop of the course, all but four FHs had procured their own computer or had secured use of a facility or neighborhood computer to bring to their workshop because they wanted to practice creating power point presentations with information they had collected during the previous months working on their CHIPs. This was not required or recommended for the course, but occurred from the students' own initiative. During the second and third workshops, facilities heads learned to overcome simple challenges like layout, diagram design, and information management to effectively present their messages. They also learned how to present publicly through stories, using their power point as a tool to add visual evidence.
