# Supplementary material for: Strengthening and Institutionalizing the Leadership and Management Role of Frontline Nurses to Advance Universal Health Coverage in Zambia
Source: Glob Health Sci Pract. 2018 Dec 27;6(4):736–46. doi: 10.9745/GHSP-D-18-00067 (PMC6370361; doi:10.9745/GHSP-D-18-00067)
Supplement: 18-00067-Nelson-Supplement1.pdf [file 18-00067-Nelson-Supplement1.pdf]

IntraHealth INTERNATIONAL
Because Health Workers Save Lives.

THE UNIVERSITY OF ZAMBIA
SERVICE AND EXCELLENCE

mPOWERING FRONTLINE HEALTH WORKERS

Primary Health Care to Communities

Leadership and Management Training for Rural Facilities Heads

Supported by
the Ministry of Health of Zambia and by Johnson & Johnson

CHIP Presentations
May 2017

CHISAMBA RURAL HEALTH CENTRE

Community Health Improvement Project

Increasing Postnatal Coverage at 6 Days at Chisamba Rural Health Centre

- Facility: Chisamba Rural Health Centre
- Facility Head: Nakaanga Wilson
- District: Chisamba
- District Supervisor: Mr. Chinyemba Maseka

Health Facility Details

Location	About 150 metres north-east of the District Health Office
Human resource	In charge 1<br>Clinical officers 2<br>Registered nurses 2<br>Enrolled nurses 4<br>EHT 1<br>Pharmacy personnel 1<br>Laboratory personnel 4<br>HIV medic 1<br>Data entry clerks 2<br>General workers 4<br>CBVs 40
Population	12 693
Services provided	MCH (static & outreach), Option B+, ART, screening, VMMC, laboratory

Improvement challenge

**Problem**	❖ **Low postnatal coverage at 6 days, 15% in 2nd Quarter 2016**
**Underlying causes**	1. Poor documentation<br>2. Low community sensitization on importance of postnatal<br>3. Postnatal service provided once in a week at the health facility<br>4. Postnatal service not integrated during outreach activities

CHIP goal

- To increase postnatal coverage at 6 days from 15% in 2nd quarter 2016 to above 50% by the end of 1st quarter 2017

Progress

Objective	Start point	End point
1. To orient 8 staff and 12 CBVs on documentation, tallying and its importance by 1st quarter 2017	Gaps in documentation of postnatal activities	❖8 staff and 12 CBVs were oriented on documentation and tallying<br>❖Improved data capturing
2. To conduct static supermarket postnatal services and visit all 11 outreach posts monthly by1st quarter 2017	Postnatal service only provided at a specific day and not integrated in the outreach activities	❖Postnatal services provided daily at the facility and all 11 outreach posts visited monthly

Progress Cont’ d

Objective	Start point	End point
3. To increase the number of monthly IEC sessions on the importance of PNC from 3 to 20 by the end of 1st quarter 2017	An average of 3 IEC sessions on postnatal were given per month at the facility.	20 IEC sessions on importance of postnatal were conducted every month in all the outreach posts and the facility

Going forward

- **Obstacles**:

- ❑ No data review meetings held.
- ❑ No staff to coordinate MCH activities

- **How we overcame the obstacles**

- ❑ A nurse assigned to coordinate MCH activities
- ❑ Mentorship and coaching
- ❑ 3 data review meetings held

Roles and Responsibilities

**Additional staff (Nurses, COs, EHT)**	**Participated in the orientation, ensured that all logistics needed were available. Provided the services and documented in the registers. Provided IEC at the facility and outreach posts**
CBVs	Participated in the orientation. Mobilized the community, provided IEC during gatherings and special events including antenatal and postnatal clinics. Mobilized the community (on dates for outreach services)
Community members	Disseminated information on postnatal services. Encouraged mothers to attend postnatal care. Mobilized the community (on dates for outreach services)

How my colleagues helped

- We shared the competencies they acquired in the training and how they applied it in their places of work and the CHIP. This encouraged me to apply the same strategies in my CHIP and place of work.
- Shared some practices my colleagues used to ensure that their indicators are above target.

CHIP monitoring

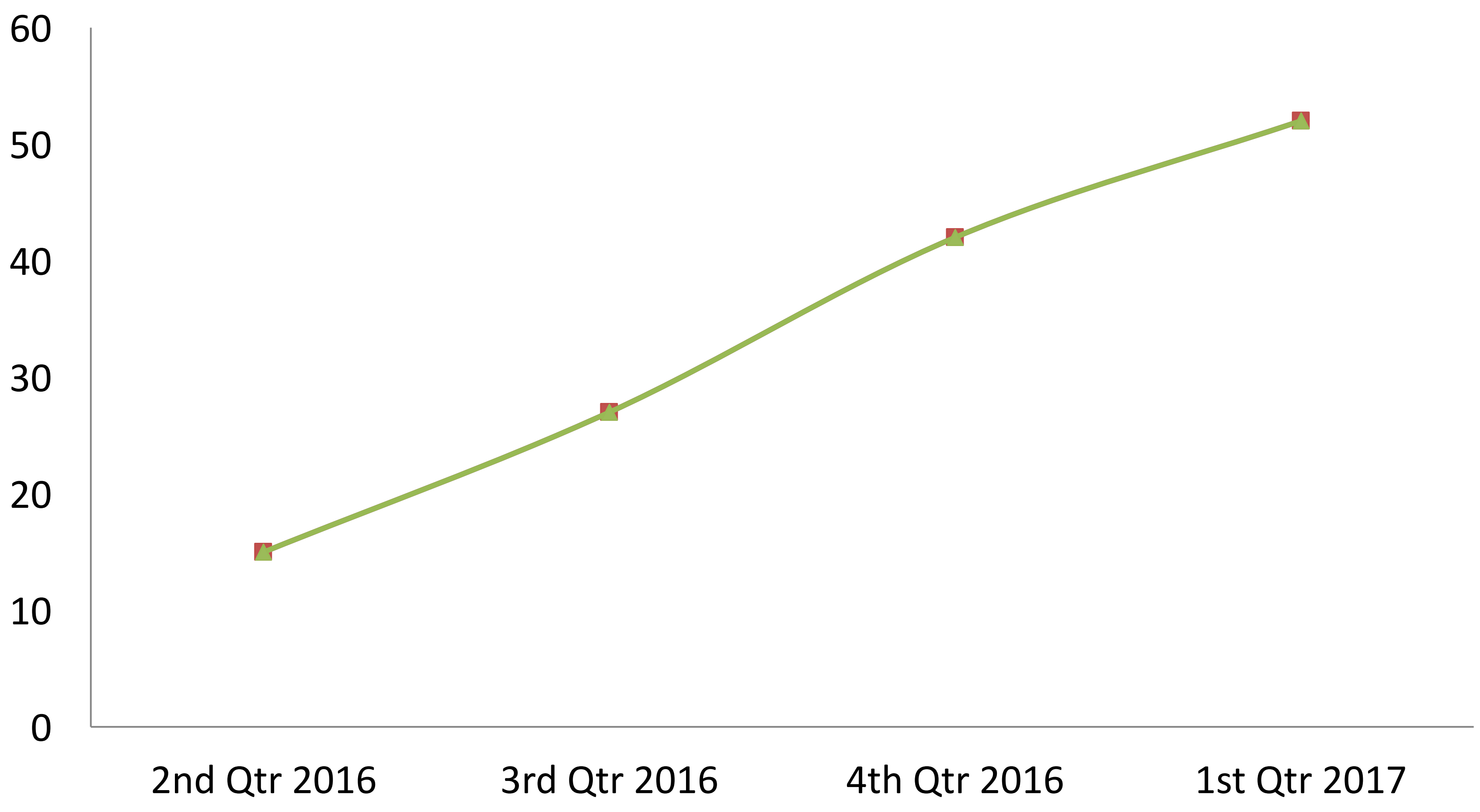

Leadership and management competencies used

- Skilled influence: made frontline team members understand the reason for staff to coordinate MCH activities.
- Built trust and accountability: Encouraged and praised frontline members for their efforts.

MPANGO RURAL HEALTH CENTRE

Community Health Improvement Project

LOW FIRST ANTENATAL VISIT

Facility: MPANGO RURAL HEALTH CENTRE
Facility Head: GIRLY HAMOONGA
District: CHONGWE
District Supervisor: VIOLET MWANZA

DETAILS OF HEALTH FACILITY

LOCATION	MPANGO IS 22KM EAST OF CHONGWE IN LUSAKA PROVINCE
HUMAN RESOURCE	• STAFF (nurses ;3, EHT 1,CO 1,CDE 2): (CO was just added to staff in Jan 2017)<br>• CBVs: 20
POPULATION	8,867
SERVICES PROVIDED	• GENERAL SCREENING<br>• ANTENATAL, POSTNATAL, F-PLANNING<br>• CHILDHEALTH AND NUTRITION<br>• ART SERVICES/OPTION B+

Improvement Challenge

Challenge:

Low 1st ANC booking at 34% against the district target of at least 80%

Underlying causes:

- Distance to the health centre
- Lack of transport at health centre
- Low male involvement

CHIP Goal:

- **To take quality ANC services as close to family as possible and reach the desired state from the current 35% ANC by May 2017.**

PROGRESS

OBJECTIVE	START POINT (October 2016)	PROGRESS TO DATE (May 2017)
1. To open and conduct outreach ANC health services in all 4 distant villages of the catchment area	0	Monthly outreach services are offered in all four distant villages. Have provided continuous services for four months (100% of goal)
3. To initiate utilization of the newly opened outreach sites.	0	94 clients have attended outreach ANC services to date (across the 4 sites) (achieved goal)
2.To increase male involvement in ANC activities from 40% to 75%	40 %	80 % (surpassed goal)

Addressing Challenges

Challenges:

- Lack of personal transport at health centre
- Motivation for CBVs

How we addressed the challenges:

- Lobbying DHO for motor bikes (still ongoing)
- Influenced to the NGO (‘Good Neighbors’) to provide a vehicle
- Appreciate hard working CBVs

ROLES AND RESPONSIBILITIES: SERVICE IMPROVEMENT OBJECTIVES 1 & 2

TO OPEN AND CONDUCT MONTHLY OUTREACH SERVICES IN DISTANT VILLAGES AND INCREASE UTILIZATION OF THESE SERVICES

FRONTLINE TEAM MEMBERS	ROLES AND RESPONSIBILITIES IN INTERVENTION (INCLUDING ACTIONS TO BE TAKEN)
**Additional Staff (nurses, EHTs, CDE)**	Active participation during ANC Services static and outreach;<br>Proper Data management on individual clients
**CBVs**	Community sensitization; Door to Door campaigns;<br>Drama sensitization<br>Head count and documentation
**Community Members / NHC, HCC, and village leadership**	Conduct Meetings in the villages to sensitize them one the importance of ANC

ROLES AND RESPONSIBILITIES: SERVICE IMPROVEMENT OBJECTIVE 3

INCREASE MALE INVOLVEMENT IN ANC ACTIVITIES FROM 40% OIN OCTOBER 2016 TO 75% BY MAY 2017

FRONTLINE TEAM MEMBERS	ROLES IN INTERVENTION
**Additional Staff (nurses, EHTs, CO ,CDE)**	Conduct clinical meetings<br>Review data monthly and qrtly
**CBVs**	Conduct interviews to ANC mothers in the community to check understanding<br>Identification of expectant mothers in the villages.
**Community Members / NHC, HCC, and village leadership**	Conduct village meetings<br>Conduct door to door sensitization

How did my colleagues in the training help me?

- **Problem solving:** Calls with colleagues of vast experience.
- **Completing workbook**: Partner calls (through the training) to discuss challenges in leadership and management exercises.
- **Motivation**: Interaction with the WHATSAPP community of practice kept me motivated each week to practice leadership and management
- **Support and Confidence:** Supervisory visits and calls with supervisor

How did I use leadership and management competencies?

- Coaching and mentoring
- Skilled Influencing
- Team building
- Clarification of roles
- Resource Management

CHIKOKA RURAL HEALTH POST

CHIP RESULTS

Community Health Improvement Project

High Teenage pregnancies

Facility: Chikoka Rural Health Post

Facility Head: Kufekisa Mulyokela

District: Kafue

District Supervisor: Mulaisho E. Shamilimo

DETAILS OF HEALTH FACILITY

LOCATION	Kafue along mungu road
HUMAN RESOURCE	• 1 Nurse<br>• 2 CHA<br>• 32 CBVs
POPULATION	3418
SERVICES PROVIDED	HCT, Reproductive Health ,Family Planning, General OPD Services, ART, Child Health Services and VMMC

Improvement Challenge

- **Challenge: High number of teenage pregnancies**
- **Underlying causes:**

➢ Peer pressure during adolescent and teenage stage

➢ Lack of knowledge on adolescent health services

➢ Sexual abuse and early marriages

➢ poverty

- **CHIP Goal:** To reduce the number of teenage pregnancies from 13% at the end of the quarter of 2016 to 8 % by the end of quarter 2 of 2017.
- **Where you are Now:** The Teenage pregnancy percentage is now at 6%.

PROGRESS

OBJECTIVE	START POINT	END POINT
1.To provide family planning services to teenagers by May 2017	Services were not being offered.	Family planning services being provided on a daily basis at the facility **(38% of adolescent accessing FP)**
2.To train adolescent CBDs and Peer educators by May 2017.	No trained adolescent CBD/Peer Educators.	Trained 6 Peer educators and 4 CBDs
3.High Teenage pregnancies	13%	6%

ROLES AND RESPONSIBILITIES: SERVICE IMPROVEMENT

FRONTLINE TEAM MEMBERS	ROLES IN INTERVENTION
**Nurse/CHAs**	-To offer FP services to teens at the center as well as in the community.<br>-To supervise and work with CBDs and Peer Educators in the community
**CBDs**	- To offer FP services in the community(i.e oral contraceptives, condoms and IEC) and report monthly
**Community Members / NHC, HCC, and village leadership**	Continuation of sensitization in the community on the benefits of teenagers accessing the FP services.

How have my colleagues in the training helped me?

- By consulting others on the challenges faced and how to solve them.
- Supervisory visit and partner call
- Do exchange visit to the best performing facility

LEADERSHIP COMPETENCIES?

Some of the leadership competencies used includes:

- **Coaching**
- **Delegation –** to the frontline team
- **Monitoring and Supervision -** of the progress so and put new approaches in progress solving
- **Team building -** through motivation and staff appraisal

MWANJUNI RURAL HEALTH POST

CHIP PROJECT

Community Health Improvement Project

CHIP TITTLE

Reduce STI incidence rate

Facility: MWANJUNI RURAL HEALTH POST

Facility Head: MERVICE MBILITU

District: CHIBOMBO

District Supervisor: MITI GERSHOM

THE HEALTH FACILITY

LOCATION	Central Province. 10 miles from Lusaka along Kabwe Road
HUMAN RESOURCE	• 1 Enrolled nurse, 1 Midwife<br>• 1 EHT<br>• 2 CHA:<br>• 12 CBVs: (HBC, TB Supporters, Malaria Agents)
POPULATION	8,914
SERVICES PROVIDED	Screening & Treatment for PHC<br>ANC, Family Planning, VCT, PMTCT (Option B+), Under-five clinic ; PNC.

Improvement Challenge

- **Challenge / Problem:**
  - **Increased cases of sexually transmitted infections**

- **Underlying causes:**
  - **Casual and multiple sex partners**
  - **No partner treatment**
  - **Abundant availability of taverns being predisposing factors**

- **CHIP Goal:**

  “To reduce incidence rate of sexually transmitted infections (from 60/1,000 cases) between October 2016 and April 2017”

CHIP GOAL

CHIP GOAL	START POINT : October 2016	END POINT: May 2017
Reduce STI incidence rate by May 2017	60/1,000	50/1,000

PROGRESS

OBJECTIVE	START POINT	PROGRESS TO DATE	END POINT
1.To establish 03 outreach STI preventive programs in the community by end of April 2017	No community programs	**Met community members,**<br>**1. STI Drama groups** have been formed and performed 1x/wk at center and 1x/ mthly at UCI post in the community;<br>**2Condom distribution** at all entry points at the facility; Also handed out at drama performances.<br>**3. Issuing of STI slips for partner notification at** facility. Increased contact tracing	03 STI preventive programs established in the Community.

PROGRESS (cont'd)

OBJECTIVE	START POINT	PROGRESS TO DATE	END POINT
2. To distribute 1.440 condoms in high risk areas per month by end of May 2017.	No condoms were being distributed	1. Identified high risk areas.<br>2. Met with tavern owners<br>3. Distributed condoms to taverns for people to have free access	1. Regular condom deliveries<br>2. Monitor and document condom refills to know if usage increases<br>3. 1,440 condoms distributed per month.

PROGRESS (cont'd)

OBJECTIVE	START POINT	PROGRESS TO DATE	END POINT
3 To reduce stock outs of condoms to zero by end of April, 2017	Monthly stock-outs of condoms.	Sourced for additional condoms from nearby facility within and from Kabwe.<br><br>Zero stock-outs of condoms since October 16	Zero stock-outs of condoms<br>Monitoring and documentation of condom supplies.

ROLES AND RESPONSIBILITIES:

OBJECTIVE #1: To establish 03 outreach STI preventive programmes in the community by end of April 2017	
**FRONTLINE TEAM MEMBERS**	**ROLES AND RESPONSIBILITIES IN INTERVENTION (INCLUDING ACTIONS TO BE TAKEN)**
**CHAs**	• Formation of STI drama groups<br>• Condom distribution
**Nurses, EHTs**	• Display of condom at all facility entry points<br>• Assigned to screen, treat, counsel, and give STI contact slips
**CBVs**	• Giving of IEC in the community.<br>• Help with condom distribution
**Community Members / NHC, HCC, and village leadership**	• Marketing the drama groups<br>• Helping with the logistics for the drama groups<br>• Health promotion

ROLES AND RESPONSIBILITIES:

INTERVENTION #2: To distribute 1,440 condoms in high risk areas per month by end of April, 2017

FRONTLINE TEAM MEMBERS	ROLES IN INTERVENTION
**CHAs**	• Monitoring of condom refill in high risk areas<br>• Identify high-risk areas<br>• Distribute condoms to high risk areas<br>• IEC
**Nurses, EHTs**	• Monitoring and documentation of condom use from facility.<br>• EHT goes with the CHA to Meet the guest house owners.<br>• IEC
**CBVs**	• Condom distribution<br>• IEC
**Community Members / NHC, HCC, and village leadership**	• Promote and advocate for condom use.<br>• IEC

ROLES AND RESPONSIBILITIES: SERVICE IMPROVEMENT OBJECTIVE 3

INTERVENTION #3:<br>. To reduce stock outs of condoms to zero by end of May 2017	
**FRONTLINE TEAM MEMBERS**	**ROLES IN INTERVENTION**
**CHAs**	Monitor and document
**Nurses, EHTs**	Ordering for restocking, Monitor and document
**CBVs**	Monitor and document
**Community Members / NHC, HCC, and village leadership**	Monitor and document

Leadership and management competencies utilised

- Monitoring and Evaluation
- Creative mobilization of resources
- Delegation of commodity ordering / refilling to a member of the staff
- Building partnership, alliance and networks
- Evidence based, Data from the registers used for analysis.

RUFUNSA RURAL HEALTH CENTRE

Community Health Improvement Project

Low Institutional Deliveries

Facility: Rufunsa Rural Health Centre

Facility Head: Florence Banda

District: Rufunsa

District Supervisor: Mr. Aliel Phiri

DETAILS OF HEALTH FACILITY

LOCATION	150 KMS FROM LUSAKA AND 1KM FROM GREAT EAST ROAD
HUMAN RESOURCE	• 2 Nurses<br>• 1 Clinical Officer<br>• 1 Environmental Health Technologist<br>• 1 Community Health Assistant<br>• 1 Data Clerk<br>• 1 Nutritionist<br>• 2 Classified Daily Employee (CDE)<br>• 25 CBVs:
POPULATION	5718
SERVICES PROVIDED	• Integrated Reproductive Health<br>• Child health and Nutrition<br>• General Out Patient Department<br>• HIV Counselling and Testing

Improvement Challenge

Problem: Low Institutional Delivery

Underlying causes:

- Lack of mother’s shelter
- 15 Inactive Smags
- Inadequate Information Education Communication (IEC) to the community on the importance of institutional deliveries

- **CHIP Goal:**

- To increase institutional deliveries by May 2017 (beginning at 23% in October 2016).

PROGRESS

GOAL AND SMART OBJECTIVES	START POINT	END POINT (AS OF PRESENTATION IN MAY 2017)
CHIP GOAL: To increase facility deliveries	23% facility deliveries	Deliveries have increased to 42% by May 2017
Smart Objective #1. To re-orient 15 Smags by end of May 2017	15 Smags existing but not oriented<br>No reports from Smags	-18 Smags re-orienteded and reporting monthly
Smart Objective #2. To engage the community to construct a Mother's shelter by 4th quarter 2017	No mother's shelter at the facility	- 20% community participation attained (contributed money, bricks, sand and man power)

ROLES AND RESPONSIBILITIES:

FRONTLINE TEAM MEMBERS	ROLES AND RESPONSIBILITIES IN INTERVENTION (INCLUDING ACTIONS TO BE TAKEN)
**Nurses/CO/EHT/CHAs**	- IEC on the importance of facility deliveries<br>- Meetings with headmen/stakeholders to educate them on issues affecting facility deliveries<br>-Monitor and supervise the CHA and Smags<br>-Making follow up to the headmen on the agreed points made during the meetings
**CBVs**	-Home visits to pregnant mothers<br>-Continuous documenting of activities monthly<br>-Door to door educating the community on the importance of facility deliveries and utilization of mother's shelter
**Community Members / NHC, HCC, and village leadership**	- Knowledgeable about the duties of a Smags in their respective communities

How did my colleagues in the training helped me?

- Do exchange visit to the best performing facility
- Consultation from our Supervisors on how best we can improve on our CHIP.
- Supervisory visit and partner call
- By learning best practices on the facility deliveries

LEADERSHIP COMPETENCIES;

- Supervision of Smags/CBVs
- Coaching and mentorship of Smags/CBV’s is on-going
- Motivation

THANK YOU.
