# Supplementary material for: Strengthening and Institutionalizing the Leadership and Management Role of Frontline Nurses to Advance Universal Health Coverage in Zambia
Source: Glob Health Sci Pract. 2018 Dec 27;6(4):736–46. doi: 10.9745/GHSP-D-18-00067 (PMC6370361; doi:10.9745/GHSP-D-18-00067)
Supplement: 18-00067-Nelson-Supplement4.pdf [file 18-00067-Nelson-Supplement4.pdf]

INSTRUMENTS FOR RETROSPECTIVE ASSESSMENT: INDIVIDUAL INTERVIEW AND FOCUS GROUP QUESTIONS

M&E QUESIONNAIRE

<u>Community Based Volunteers</u>

Zambian Certificate Training for Leadership, Management, and Practice For Heads of Rural Health Posts and Rural Health Centers

July / August 2017

Introduction

"Hello, we are representing the MoPH, and we are here to review changes that (patients/ staff/ volunteers, etc) have seen in the quality of services over the past six months. So, we will be going to ask you about changes you have seen services here and find out if any have improved. Your answers are anonymous. Some you will answer as a group, and some we ask you individually. Please do be honest and thorough. We will not know if we are improving services well unless we have honest and thorough answers."

Does everyone here agree to participate in this focus group, understanding that they are not coerced in any way and are free to decline participation at any time. (***Give time for all to agree; please excuse anyone who is not able to agree)***

Does each person here understand that their names are not recorded and their responses will not be shared with the other staff or community members and that all responses are put into a mix with many other responses so are not attributable to any one person. (***Give time for all to agree; please excuse anyone who is not able to agree)***

Then continue:

[Note for these questions, the data collectors should say how many people were in the discussion group, and document how many people said (same; better; and worse) for each question]

1. What are your roles in the frontline team?
2. How has the head nurse helped them to fit into the frontline team?
3. Does the head nurse in the facility help you do your job in the best way possible?
    a. If so, how?
    b. If so, is she/he do anything differently to help you than she did six months go?
4. Does the nurse recognize the volunteers who provide quality services?
    a. If so, how?
    b. IF so, does he/she appreciate you more than he/she did six months ago?
        i. If so, how?
5. How do you coordinate your work with the other staff and volunteers?
    a. Does the nurse help you to do that?
    b. If so, how?
6. Do the head nurse assign tasks to the volunteers and the staff to help you improve the way you work?
    a. If yes, How?
    b. If yes, is it different now or the same as it was six months ago?
    c. If different, How so?
7. Have you learned anything about quality improvement from the head nurse?
    a. If yes, please explain
8. Do you interact differently with the NHC members now than you did six months?
    a. If yes, please explain.
9. Have the families in the community seen a change in the way that you and the others provide care?
    a. If yes, please explain. (what change? How is it different? What would the community notice?)
    b. Is it different to what it was 6 months ago?
10. What was the CHIP and what role did you play in its implementation?

11. What have you learnt about quality improvement from the CHIP?

12. How do you work as a team at this facility? Is it different from what it was 12 months ago?

M&E QUESIONNAIRE

CHA

Zambian Certificate Training for Leadership, Management, and Practice
For Heads of Rural Health Posts and Rural Health Centers

July / August 2017

Introduction

"Hello, we are representing the MoPH, and we are here to review changes that (patients/ staff/ volunteers, etc) have seen in the quality of services over the past six months. So, we will be going to ask you about changes you have seen services here and find out if any have improved. Your answers are anonymous. Some you will answer as a group, and some we ask you individually. Please do be honest and thorough. We will not know if we are improving services well unless we have honest and thorough answers."

Does everyone here agree to participate in this focus group, understanding that they are not coerced in any way and are free to decline participation at any time. (***Give time for all to agree; please excuse anyone who is not able to agree)***

Does each person here understand that their names are not recorded and their responses will not be shared with the other staff or community members and that all responses are put into a mix with many other responses so are not attributable to any one person. (***Give time for all to agree; please excuse anyone who is not able to agree)***

Then continue:

[Note for these questions, the data collectors should say how many people were in the discussion group, and document how many people said (same; better; and worse) for each question]

1. How is the head nurse leading the staff and volunteers?
2. Have you learned any new clinical skills from the facility head since December?
    a. If yes, please explain.
3. Have you learned any non-clinical skills from the facility head since December?
    a. If yes, please explain
4. How would you describe your facility head's management style? (***whatever they say is fine- do not lead them)***
5. Does the head nurse coach you?
    a. If so, how?
    b. If so, has the coaching style changed in the last six months?
    c. If so, please explain
6. Does the head nurse coach others?
    a. If so who?
    b. If so, has the coaching style changed in the last six months?
    c. If so, please explain
7. Do you coach anyone?
    a. other staff?
    b. Volunteers?
    c. Patients?
    d. If you coach others, has the coaching style changed in the last six months?
    e. If so, please explain
8. Does the head nurse delegate responsibilities or tasks to you?
    a. If so, what?
    b. If so, is this different from how he/she delegates now and how he/she used to delegate six months ago?
    c. If yes, please explain.
9. Has the nurse helped you and the other CHA to integrate into the team?
    a. If yes, does the nurse do anything differently now to help you since he/she went through his /her training?
10. Does your head nurse help you do your job better?
    a. Is this any different from what it was 6 months ago?
11. Have the families in the community seen a change in the way that you and the others provide care?
    a. If yes, please explain. (what change? How is it different? What would the community notice?)
    b. If yes, when did that change take place?
12. What was the CHIP and what role did you play in its implementation?
13. What have you learnt about quality improvement from the CHIP?
14. How do you work as a team at this facility? Is it different from what it was 12 months ago?

M&E QUESIONNAIRE

CHA

Zambian Certificate Training for Leadership, Management, and Practice
For Heads of Rural Health Posts and Rural Health Centers

July / August 2017

Introduction

"Hello, we are representing the MoPH, and we are here to review changes that (patients/ staff/ volunteers, etc) have seen in the quality of services over the past six months. So, we will be going to ask you about changes you have seen services here and find out if any have improved. Your answers are anonymous. Some you will answer as a group, and some we ask you individually. Please do be honest and thorough. We will not know if we are improving services well unless we have honest and thorough answers."

Does everyone here agree to participate in this focus group, understanding that they are not coerced in any way and are free to decline participation at any time. (***Give time for all to agree; please excuse anyone who is not able to agree)***

Does each person here understand that their names are not recorded and their responses will not be shared with the other staff or community members and that all responses are put into a mix with many other responses so are not attributable to any one person. (***Give time for all to agree; please excuse anyone who is not able to agree)***

Then continue:

[Note for these questions, the data collectors should say how many people were in the discussion group, and document how many people said (same; better; and worse) for each question]

1. How is the head nurse leading the staff and volunteers?

2. Have you learned any new clinical skills from the facility head since December?
    a. If yes, please explain.
3. Have you learned any non-clinical skills from the facility head since December?
    a. If yes, please explain
4. How would you describe your facility head's management style? (***whatever they say is fine- do not lead them)***
5. Does the head nurse coach you?
    a. If so, how?
    b. If so, has the coaching style changed in the last six months?
    c. If so, please explain
6. Does the head nurse coach others?
    a. If so who?
    b. If so, has the coaching style changed in the last six months?
    c. If so, please explain
7. Do you coach anyone?
    a. other staff?
    b. Volunteers?
    c. Patients?
    d. If you coach others, has the coaching style changed in the last six months?
    e. If so, please explain
8. Does the head nurse delegate responsibilities or tasks to you?
    a. If so, what?
    b. If so, is this different from how he/she delegates now and how he/she used to delegate six months ago?
    c. If yes, please explain.
9. Has the nurse helped you and the other CHA to integrate into the team?
    a. If yes, does the nurse do anything differently now to help you since he/she went through his /her training?
10. Does your head nurse help you do your job better?
    a. Is this any different from what it was 6 months ago?
11. Have the families in the community seen a change in the way that you and the others provide care?
    a. If yes, please explain. (what change? How is it different? What would the community notice?)
    b. If yes, when did that change take place?
12. What was the CHIP and what role did you play in its implementation?
13. What have you learnt about quality improvement from the CHIP?
14. How do you work as a team at this facility? Is it different from what it was 12 months ago?

M&E QUESIONNAIRE

<u>NEIGHBORHOOD HEALTH COMMITTEES (NHCs) and</u>
<u>HEALTH CENTER COMMITTEEES (HCCs)</u>

Zambian Certificate Training for Leadership, Management, and Practice
For Heads of Rural Health Posts and Rural Health Centers

July/August 2017

Introduction

"Hello, we are representing the MoPH, and we are here to review changes that (patients/ staff/ volunteers, etc) have seen in the quality of services over the past six months. So we will are going to ask you about changes you have seen services here and find out if any have improved. Your answers are anonymous. Some you will answer as a group, and some we ask you individually. Please do be honest and thorough. We will not know if we are improving services well unless we have honest and thorough answers."

Does everyone here agree to participate in this focus group, understanding that they are not coerced in any way and are free to decline participation at any time. (***Give time for all to agree; please excuse anyone who is not able to agree)***

Does each person here understand that their names are not recorded and their responses will not be shared with the other staff or community members and that all responses are put into a mix with many other responses so are not attributable to any one person. (***Give time for all to agree; please excuse anyone who is not able to agree)***

Then continue:

[Note for these questions, the data collectors should say how many people were in the discussion group, and document how many people said (same; better; and worse) for each question]

1. Do you contribute to strategies to solve community health problems?
    a. If yes, do you do things differently now than you did six months ago?
    b. If yes, please explain what you do differently and why.
2. Does the head nurse bring the whole team together to promote better health?
    a. If yes, then How?
    b. If yes, then is that different or the same as six months ago?
    c. If different, please give examples
3. Does your community feel that quality services are delivered at the health facility?
    a. If yes, how do you know (please give specific examples)
    b. If yes, have they improved, remained the same, or even less than they six months ago?
4. Do CBVs provide quality services to the households?
    a. If yes, are they providing better services than they were six months ago, the same level, or a lower level of service than six months ago?
    b. If better, what has changed?
5. Does the team work well together?
    a. If yes, is the coordination better, the same, or less than it was six months ago?

<u>CHIP Specific questions here.</u>

6. What was the CHIP in your catchment area (to see if they know about it?)
7. How did you each contribute to the CHIP?
8. Did the nurse in charge of your facility help to lead the CHIP?
    a. If yes, please explain
9. Did you learn anything about Quality Improvement from the CHIP?
    a. If yes, please explain
10. Was the CHIP successful?
    a. If yes, how?
11. Will you carry out similar types of activities together in your community?
    a. If yes, please explain.
12. Have the NHC members learned anything new in the last six months from the head nurse?
13. Are the staff or volunteers doing anything differently in the last 6 months?
    a. If yes, please explain

M&E QUESIONNAIRE

<u>FACILITY STAFF</u>

Zambian Certificate Training for Leadership, Management, and Practice
For Heads of Rural Health Posts and Rural Health Centers

July / August 2017

Introduction

"Hello, we are representing the MoPH, and we are here to review changes that (patients/ staff/ volunteers, etc) have seen in the quality of services over the past six months. So, we will be going to ask you about changes you have seen services here and find out if any have improved. Your answers are anonymous. Some you will answer as a group, and some we ask you individually. Please do be honest and thorough. We will not know if we are improving services well unless we have honest and thorough answers."

Does everyone here agree to participate in this focus group, understanding that they are not coerced in any way and are free to decline participation at any time. (***Give time for all to agree; please excuse anyone who is not able to agree)***

Does each person here understand that their names are not recorded and their responses will not be shared with the other staff or community members and that all responses are put into a mix with many other responses so are not attributable to any one person. (***Give time for all to agree; please excuse anyone who is not able to agree)***

Then continue:

[Note for these questions, the data collectors should say how many people were in the discussion group, and document how many people said (same; better; and worse) for each question]

1. How is the head nurse working with the staff and volunteers? How would you describe your facility head's management style?
    a. Is this differently or the same as he/she did six months ago.
    b. If differently, please explain.
2. Does the head nurse delegate responsibilities or tasks to you?
    a. If so, what?
    b. If so, is this different or the same from how he/she used to delegate?
3. If different, please explain.
4. Have you learned any new clinical skills from the facility head since December?
    a. If yes, please explain.
5. Have you learned any non-clinical skills from the facility head since December?
    a. If yes, please explain

6. Does the head nurse coach you?
    a. If so, how?
    b. Is this any different than he/she coached you before?
    c. If yes, please explain.
7. Does the head nurse coach others?
    a. If so who?
    b. Is this any different than he/she coached these others before?
    c. If yes, please explain.
8. Do you coach anyone?
    a. other staff?
    b. Volunteers?
    c. Patients?
    d. Is your coaching any different than it was six months ago?
    e. If yes, please explain

9. What was the CHIP and what role did you play in its implementation?

10. What have you learnt about quality improvement from the CHIP?

11. How do you work as a team at this facility? Is it different from what it was 12 months ago?

M&E QUESIONNAIRES

Nurse In-charge

Zambian Certificate Training for Leadership, Management, and Practice
For Heads of Rural Health Posts and Rural Health Centers

July / August 2017

1. Comment on what you learnt and how you have practiced what you learnt in the following modules giving examples: Mention any new skills you may have picked up:
   Coaching,
   Strategic Planning etc.

2. Comment on the materials you were provided with for learning such as Printed modules, WhatsApp, UNZA Mobile etc. How did they aid in your learning?

3. How has this training made adifference on the following (Y/N):?
   a. Delegation of duties/activities
   b. Team building
   c. Improving quality of services
   d. Increasing number of patients accessing services
   e. Increasing your self- confidence

4. What were the roles of your supervisors that made this training effective? Comment on your interaction with your supervisors.

5. What new information have you gathered in the last year that has improved your clinical practice?

6. What parts of this course will you strongly recommend?
7. What parts of the course need improvement?
8. How can we improve the course for the next cohort?

9. Are the medicines/commodities that you are prescribed available for you to pick up?

   a. Has the medicine supply improved, stayed the same, or worsened over the last six months?
   b. Please explain (giving specific examples
9. What was your CHIP?

10. Briefly describe the steps taken to formulate the CHIP and how it was implemented in your Health facility.

Mentors participating in the training

1. Did you see services improve, stay the same, or worsen in the facilities where the nurse in charge was participating in the UNZA PHC2C training?
   a. If so, please describe (be specific)
   b. If so, did the communities involved see changes (please explain)
   c. If so, did the staff and volunteers see changes?
2. Did you see a change in the leadership practices and/or management practices of the nurse in charge taking the course?
   a. Leadership? Give specific examples of each of the nurses you work with.
   b. Management? Give specific examples again for each nurse
3. Did your relationship change with the nurses in the facilities in this training?
   a. Please explain
   b. Please explain how these are or are not different than your relationship with the other facilities heads you supervise.
4. Did you change your supervision style?
   a. If so, then please explain (be specific)
   b. If so, was this change an improvement?
   c. If so, will you engage these changes with other facilities?
5. Do you recommend that this course be offered to other facilities heads?
6. Do you think that this training helps the facilities heads provide more value and/or support to the district managers?
   a. Please explain your answer
7. Do you like the role that you played in the training?
   a. Please explain
   b. What would you like to do differently?
8. What improvements do you recommend in the training?
9. Do you feel that this training is valuable for other facilities? Districts?
10. What do you want others to know about this course?
