# Supplementary material for: Strengthening and Institutionalizing the Leadership and Management Role of Frontline Nurses to Advance Universal Health Coverage in Zambia
Source: Glob Health Sci Pract. 2018 Dec 27;6(4):736–46. doi: 10.9745/GHSP-D-18-00067 (PMC6370361; doi:10.9745/GHSP-D-18-00067)
Supplement: 18-00067-Nelson-Supplement2.pdf [file 18-00067-Nelson-Supplement2.pdf]

CLMP Participating Facilities, with Community Health Improvement Project (CHIP) Goals and Results

Province/ District	Facility	Nurse In-Charge	Staff*	CBVs	Population Served	CHIP Goal	CHIP Results
Central/ Chisamba	Chisamba RHC	Wilson Nakaanga	MW-1, N-6, EHT-1, LT-1, PT-1, CDE-2	60	12,693	Increase postnatal coverage at 6 days	Achieved: From 15% in March 2016 to 50% by March 2017.
Central/ Chisamba	Chikonkomewe Mulungushi RHC	Nathan Hamooya	MW - 2, N- 1, EHT - 1, CHA - 3, CDEs - 1	60	12,656	increase the % of under ones fully immunized	Achieved: Began at 6% in June of 2016 and reached 80% by June of 2017
Central/ Chisamba	Mwapula RHP	Sydney Chazula	CO - 1, CHA - 2, CDE - 1	47	6,521	Increase facility deliveries	Achieved: Began at 29% in July 2016 and reached 47% in May of 2017.
Central/ Chisamba	Momboshi RHP	Milani Mayoba	MW -1, N-1, EHT - 1	65	14,922	Increase facility deliveries	Achieved: Began at 40% in 2nd quarter 2016, and reached 70% by the end of the first quarter 2017
Lusaka/ Chongwe	Chongwe RHC	Mary Munthali Kahula	MW- 2, N-8, EHT - 1, LT - 3, PT - 4, CDEs - 4, COs - 4	35	17,328	Increase the percentage of HIV/AIDS exposed infants that are tested are tested for HIV at 18 months	Achieved: Began at 35% in October 2016 and achieved 55% at end of second quarter 2017 (June)
Lusaka/ Chongwe	Kapete RHP	Joseph Chakwira	CHA-1	9	1,425	Provide Patient STI counseling and testing	Achieved: Began at 0% in October 2016 and ended at 75% in May 2017
Lusaka/ Chongwe	Shiyala RHP	Margaret Mataka	CHA-1	18	2,457	Increase early enrollment of pregnant women in ANC	Achieved: Began at 30% in June 2016 and reached 80% in June of 2017

Province/ District	Facility	Nurse In-Charge	Staff*	CBVs	Population Served	CHIP Goal	CHIP Results
Lusaka/ Chongwe	Mpango RHC	Girly Hamoonga	N-2, CDE-2	20	9,005	ANC services as close to family as possible and reach the desired state of early enrollment of all pregnant women in ANC	Achieved: Began at 35% early ANC enrollment in October 2016 and achieved 80% by June 2017
Lusaka/ Kafue	Kafue Mission RHC	Grace Mwamba	MW- 6, N - 1, EHT - 1, LT - 1	68	10,484	Increase the percentage of women attaining FANC (focused antenatal care)	Achieved: Began at 57% in October 2016 and reached 70% by June 2017
Lusaka/ Kafue	Chikoka RHP	Kufekwa Kufekisa Mulyokela	N-1, CDE-2, CHA-2	33	3,394	Reduce the number of teenage pregnancies from 13% at the end of the quarter of 2016 to 8 % by the end of quarter 2 of 2017	Almost Achieved: Began at 13% in June 2016 and ended at 8% by end of May 2017
Lusaka/ Kafue	Kabweza RHP	Albert Chiba	N-1, CDE-2, CHA-2	45	3,120	Reduce the number of diarrhoeal cases per month	Achieved: Began at a monthly average of 50 cases in October 2016 to 16 cases in April of 2017
Lusaka/ Kafue	Muguramено RHP	Rosemary Chimwanga	MW-1, CDE-2, CHA-2	36	3,080	To increase 1st ANC coverage below 14 weeks	Achieved: Began at 35% in October 2016 and reached 62% in May 2017
Central/ Chibombo	Shimukuni RHC	Gertrude Habeenzu Banda	N- 1, MW-1, EHT - 1, CDE - 1	30	8,138	Increase the testing of HIV exposed infants	Achieved: Began at 60% in March of 2016 and

Province/ District	Facility	Nurse In-Charge	Staff*	CBVs	Population Served	CHIP Goal	CHIP Results
						(HEI) at 18 months	reached 83% in April of 2017.
Central/ Chibombo	Kabangalala RHC	Mercy Akende	N-1, CDE-1	4	6,023	Increase facility deliveries	Achieved: Began at 40% in September 2016 and reached 56% by May 2017
Central/ Chibombo	Mwanjuni RHP	Mervice Mbiliti	CDE-1, EHT-1, CHA-2	12	8,512	Reduce STI incidence rate	Achieved: Began at 6% in October 2016 and reached 5% in May 2017
Central/ Chibombo	Chamakubi RHP	Markson Subeti	CDE-1, CHA-2	n/a	6,314	Increasing Post Natal Care services at 6 weeks	Achieved: Been at 55% in March 2016 and reached 84% in March 2017
Lusaka/ Rufunsa	Rufunsa RHC	Florence Banda	CDE-2, EHT-1, CHA-1	6	5,463	To increase institutional deliveries	Achieved: Began at 23% in October 2016 and reached 42% in May 2017
Lusaka/ Rufunsa	Nyamanongo RHP	Agness Mweemba	CHA-2	6	3,215	Increase retention on PNC	Achieved: Began at 30% in June 2016 and reached 50% in June 2017

*Midwife (MW), Nurse (N), Clinical Officer (CO), Environmental Health Technician (EHT), Lab Technician (LT), Pharmacy Technician (PT), Classified Daily Employee (CDE), Community-Based Volunteers (CBVs)
